# Pass/Fail Versus Tiered Grades and Academic Performance in Undergraduate Medical Education: Crossover Study

**DOI:** 10.2196/74975

**Published:** 2025-12-02

**Authors:** Boris Modrau, Karina Frahm Kirk, Sinan Mouaayad Abdulaimma Said, Carsten Reidies Bjarkam, Lone Sunde, Jacob Bodilsen, Jakob Dal, Jette Kolding Kristensen, Jeppe Emmersen, Mike Bundgaard Astorp, Stig Andersen

**Affiliations:** 1 Department of Clinical Medicine, Faculty of Health, Aalborg University, Selma Lagerløfs Vej 249Gistrup, 9940, Denmark; 2 Stroke Unit, Department of Neurology, Aalborg University Hospital, Aalborg, Denmark; 3 Faculty of Health, Aalborg University, Gistrup, Denmark

**Keywords:** exam assessment methods, pass/fail, tiered grade, evaluation methods, undergraduate, medical education, exam, academic achievement

## Abstract

**Background:**

The impact of Pass/Fail or Tiered grade assessment for exams in undergraduate medical education has caused much debate, but there is little data to inform decision-making. The increasing number of medical schools transitioned to a Pass/Fail assessment has raised concerns about medical students’ academic performance. In 2018, during the undergraduate medical curriculum reform at the Faculty of Medicine, Aalborg University changed some exams from Pass/Fail to Tiered grade and vice versa for other exams. These changes provide an opportunity to evaluate the different assessment forms.

**Objective:**

This study aimed to evaluate medical students’ academic performance at the final licensing exam in relation to the exam grading principle.

**Methods:**

This single-center cohort study at Aalborg University Medical School, North Denmark Region, assesses the change from 2-digit Tiered grade to Pass/Fail evaluation and vice versa of undergraduate medical students’ exams after the 4th and 5th year clinical training modules from Autumn 2015 through Spring 2023. The primary outcome was (1) the average grades at the final licensing exam and (2) the number of students failing exams during the previous two years.

**Results:**

Among the total of 7634 exams, 7164 4th and 5th year clinical training exams were included in the comparisons, of which 3047 (42.5%) were Pass/Fail exams and 4117 (57.5%) were Tiered grade exams. The frequency of students failing exams was 3.3% (n=101/3047) at Pass/Fail and 1.97% (81/4117) with Tiered grade exams (*P*<.001). This difference was leveled out when counting the near-failure tiered grade as Fail. Tiered grade exams did not differ between semesters (*P*=.99) nor show a time trend at the 4th year (*P*=.66). The final licensing exam grades were unaltered (*P*=.47).

**Conclusions:**

Contrary to our expectation, Pass/Fail exams exhibited a higher fail rate compared to Tiered grade exams without lowering the final academic performance. These results suggest that a shift from Tiered grades to Pass/Fail assessment redirects the focus from rewarding high performance to ensuring standards are maintained among underperforming students.

## Introduction

The importance of exams for undergraduate medical education cannot be overestimated [1], and scores in assessments is a delicate matter gaining much attention [2]. Pass/Fail grading has the benefits of reduced stress, enhanced well-being, supporting a less competitive learning environment with a greater focus on learning [3,4]. This is of interest as a narrative review reported a high number of medical students experienced test anxiety [5]. Furthermore, Tiered grading systems do not consistently correlate with future academic performance [6], which may support a transition towards Pass/Fail grading. Accordingly, a number of medical schools have transitioned to Pass/Fail and the National Board of Medical Examiners in the United States has changed reporting the Step 1 United States Medical Licensing Examination (USMLE) from a scored test to a Pass/Fail assessment.

While these changes support a learning environment in which students learn to become excellent physicians rather than exam-experts, the impact on academic performance has been a major concern. Even though the shift to Pass/Fail grading in the USMLE Step 1 resulted from good intentions, it might have had unintended consequences. Exam-related anxiety might even increase, as medical students now only have one chance to obtain a top score on Step 2 of the USMLE [7]. This might have consequences in the selection for residency programs as top scores have been used as a predictor for a successful residency [8] and as an important selection parameter [9]. However, presumptions and preconceptions may blur a clearer view on grading at exams, and data are needed to support decision-making.

Exam outcomes are readily available as all assessments can be retrieved from Digital Exam, thus supporting quality control by regularly evaluating study outcomes. The curriculum reform of 2018 at Aalborg University Medical School changed assessment at exams between Pass/Fail and Tiered grades while retaining learning objectives, learning principles, and module organization for the 4th and 5th year. Study Board discussions and reflections among a study lead group, leading up to the decision of revising grading principles, inspired the present evaluation, as the changed grading principles provided a unique opportunity for empirically grounded evaluation of the shift from Tiered grades to Pass/Fail assessment in undergraduate medical education.

The aim of this study was to evaluate the shift from Tiered grades to Pass/Fail assessment in undergraduate medical education by comparing the outcome of Tiered grades and Pass/Fail grading at exams at the 4th and 5th year from a large, longitudinal dataset with a crossover design, and to evaluate medical students’ academic performance at the 6th year final licensing exam.

## Methods

**Figure 1.**
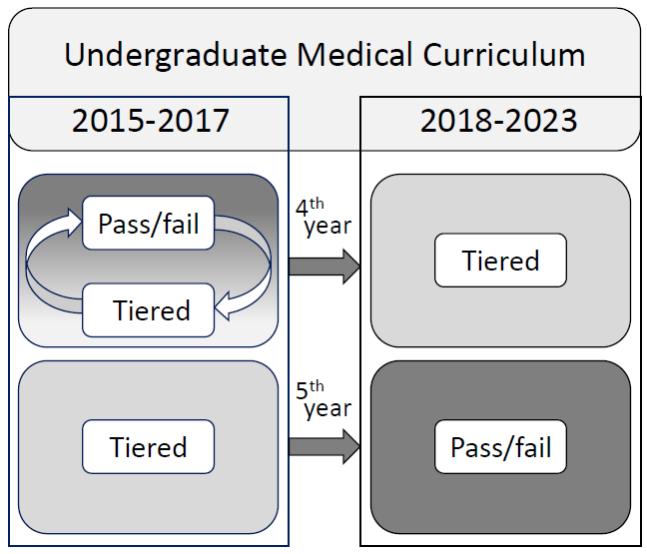
Change of grading in the exams with the 2018 reform of the undergraduate medical curriculum at Aalborg University.

Medical undergraduate education in Denmark is a six-year program split into a Bachelor’s program of three years focusing on basic sciences, and a Master’s program of three years with a clinical focus. The transformation from basic sciences into clinically based learning included the medical students entering the clinical environment from day 1 in their 4th year. The first clinical year, being the 4th year in the undergraduate medical education, alternated between Pass/Fail at winter exams and Tiered grades at summer exams until the 2018 curriculum reform (Figure 1).

Year-4 exam assessments (upper, left) changed from alternating Pass/Fail and Tiered grades to Tiered grades for all exams (upper, right). Year-5 exam assessments changed from Tiered Grades (lower, left) into Pass/Fail (lower, right).

The 2018 reform [6] changed all 4th year exam assessments to use Tiered grades with the aim of supporting students’ awareness of their academic level when entering the clinical years. Year-4 exams were oral exams with patients for Internal Medicine and Surgery, and a multiple choice questionnaire for Pathology (Table 1).

**Table 1.** Overview of exam type, scoring, and number of examinations split by the two time periods before and after the 2018 curriculum reform.

Study year and specialty	Examination type	Scoring				Number of examinations		Total number
		2015‐2017		2018‐2023		2015‐2017	2018‐2023	2015‐2023
		Autumn	Spring	Autumn	Spring			
4th								
Surgery	Oral patient based^a^	Pass/ Fail	Tiered grades	Tiered grades	Tiered grades	327	593	920
Internal Medicine	Oral patient based^a^	Pass/ Fail	Tiered grades	Tiered grades	Tiered grades	327	602	929
Pathology	Multiple-choice questionnaire^b^	Pass/ Fail	Tiered grades	Tiered grades	Tiered grades	334	1204	1538
5th								
Gynecology/ Obstetrics	Oral case based	Tiered grades	Tiered grades	Pass/ Fail	Pass/ Fail	209	425	634
Neurology/ Neurosurgery	Oral case based	Tiered grades	Tiered grades	Pass/ Fail	Pass/ Fail	201	421	622
Ophthalmology	Oral case based	Tiered grades	Tiered grades	Pass/ Fail	Pass/ Fail	202	429	631
Otorhino-laryngology	Oral case based	Tiered grades	Tiered grades	Pass/ Fail	Pass/ Fail	203	422	625
Pediatrics	Oral patient based^c^	Tiered grades	Tiered grades	Pass/ Fail	Pass/ Fail	209	426	635
Psychiatry	Written case based	Tiered grades	Tiered grades	Pass/ Fail	Pass/ Fail	203	427	630
6th								7164
Final licensing exam	Oral patient based	Tiered grades	Tiered grades	Tiered grades	Tiered grades	^—d^	470	470
								7634

a Students handed in a portfolio of 12 admission papers to be granted access to the oral exam.

b MCQ, Multiple Choice Questionnaire of a case and photo with 4‐5 options for an answer.

c The patient could be swapped for a case.

d Not applicable.

In the succeeding 5th year, students were expected to have adjusted to clinically based learning, and the 2018 reform changed all exam assessments from Tiered Grades to Pass/Fail for all modules (Figure 1). The aim of switching to Pass/Fail evaluation for all exams during the 5th year was to support the students’ focus on learning to be a medical doctor rather than studying for exams. The year-5 exams were case-based oral exams for Gynecology/Obstetrics, Neurology, Neurosurgery, Ophthalmology, and Otorhinolaryngology, oral exam with patients for Pediatrics, and a written stepwise case-based exam for Psychiatry (Table 1).

The final 6th year used Tiered Grades throughout the observation period (Table 1) for this study to comply with legislation for final medical exams in Denmark.

Admission criteria in Denmark are set by legislation, and they rely heavily on average grade points from high school. Thus, the admission principle was unchanged throughout the study period, making the students entering medical school on similar terms during the years of the present study.

At Aalborg University Medical School, problem-based learning (PBL) is the learning principle practiced during the three years of the Bachelor’s program, which feeds into the Master’s program’s patient-based learning [10]. The first two years of the Master’s program comprises a series of clinical placements in the morning, supported by patient-based, theory-led small-group PBL-tutorials engaging in discussions with their tutors in the afternoon. Medical students are referred to as apprentice-doctors to support a learning focus on what is needed to be a doctor at the basic level. The final year is theory based aiming to foster deep learning when revisiting all aspects of medicine from the previous five years, thus supporting a spiral curriculum.

The learning objectives, module organization, and teaching principles were unaltered throughout the study period. The academic staff did not change during the years of the study, and examiners were unaware of the present evaluation.

The 4th year consisted of internal medicine, surgery, and pathology. The 5th year comprised Pediatrics, Gynecology/Obstetrics, Neurology, Neurosurgery, Otolaryngology, Ophthalmology, and Psychiatry. All exams for all modules were included in the present evaluation, and the modules were unaltered except for the grading principles. The subgroup with alternating evaluation and the subgroup with permanent change from Tiered Grades to Pass/Fail grading were analysed. Licensing exams in the 6th year were included in the analysis for evaluation of final academic performance.

The Danish grading scale has seven steps, and it is almost comparable to the European Credit Transfer and Accumulation System (ECTS) scale A-F: 12; 10; 7; 4; 02; 00; and −03. While 02 is a Pass, it is the lowest of five pass grades and represents near-fail. Conversely, 00 and −03 represent a clear Fail without modification, and the delineation of failing exams is quite distinct. Thus, Pass/Fail was computed from the 2-digit Tiered grade exam assessments.

### Statitical analysis

Results are reported in percent and in the crude numbers, and comparisons were performed using the *χ*^2^ test for comparison of proportions, the Kruskal-Wallis test for comparing numerical grades between semesters, and the Kendall test to test for trend in Tiered grade exams over time. Internal validity was explored by monitoring grade point averages at the 4th year exams using tiered grades. As student cohorts, examiners, and formats remained stable across the years of the study, no additional adjustment for confounders was performed. Student-level variables were not available for the analysis, but in accordance with unaltered entry criteria, age and gender distributions were similar across the study years. Statistical analyses were performed using SPSS Statistics for Windows (version 13.0; IBM Corp).

### Ethical Considerations

All data on grades was retrieved and reviewed for quality assurance in medical education, and grades were reported anonymously to Aalborg University using a digital solution (Digital Eksamen, Arcanic A/S, Copenhagen). The need for ethical approval or informed consent was waivered for anonymous data retrieved for quality assurance. Still, the presented research meets the recommendations by The Danish Code of Conduct for Research Integrity, with the primary material being digital raw data provided as an anonymous list of grades. Participants or the public were not involved in the design, conduct, reporting or dissemination plans for this study.

## Results

Our evaluation comprises 7634 exams for 16 semesters from the Autumn of 2015 through Spring of 2023. In total, 7164 exams with 66 exam sessions prior to, and 78 exam sessions following the 2018 curriculum reform, were included in the comparisons during the 4th and 5th year. Of these 3047 (42.5%) were Pass/Fail exams and 4117 (57.5%) were Tiered grade exams. Additionally, 470-Tiered grade final licensing exams at the 6th year were included for evaluation of the final academic performance.

**Figure 2.**
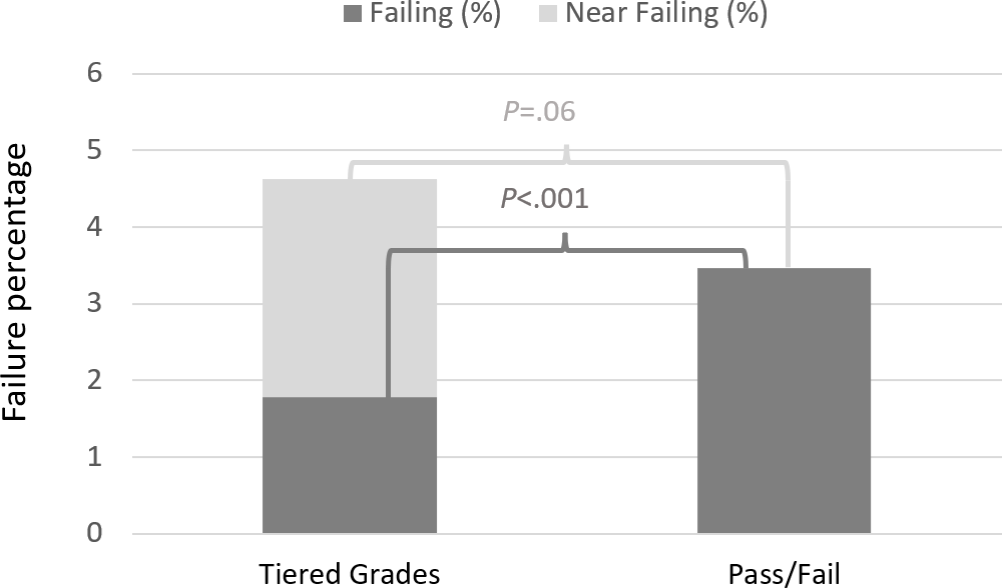
Frequency of students failing exams depending on the type of examination.

The overall frequency of students failing exams is illustrated in Figure 2.

The frequency of students failing exams (dark gray bars) was 3.3% (n=101/3047) when using Pass/Fail and 2.0% (81/4117) with Tiered grade evaluations (*χ*^2^ test*, P*<.001). This difference was leveled out (*χ*^2^ test, *P*=.055) when including the near-failure Tiered grade as fail (light gray bar).

In the subgroup of 988 examinations alternating between Tiered grade and Pass/Fail (upper left box in Figure 1), the frequency of students failing exams was 1.8% (9/497) with Pass/Fail and 0.8% (4/491) with Tiered grade exams (*χ*^2^ test, *P*=.20). In the subgroup of 5th year students switching permanently from Tiered grade to Pass/Fail (lower boxes in Figure 1), the frequency of students failing exams was 3.6% (92/2550) with Pass/Fail and 1.1% (13/1227) with Tiered grade assessments (*χ*^2^ test, *P*<.001).

The level of grades at the Tiered grade exams did not differ between semesters (Kruskal-Wallis test, *P*=.99) nor show a time trend at the 4th year (Kendall test, *P*=.66). The average grades of the 6th year final licensing exam (Kendall test, *P*=.47) and SD of about 3 were unchanged over the entire study period (Table 2).

**Table 2.** Average grades at year-4 before and after the 2018 reform and the final licence exams at year 6.

Years	Year-4 grades, mean (SD)	Year-6 grades, mean (SD)
2016	8.8 (2.7)	—
2017	8.5 (2.8)	—
2018	8.5 (2.9)	—
2019	8.4 (2.9)	9.3 (2.8)
2020	8.5 (2.9)	8.5 (3.0)
2021	8.3 (3.0)	9.1 (2.8)
2022	8.3 (3.2)	8.4 (3.4)
2023	8.6 (3.0)	8.7 (3.1)

The Danish Tiered Grade scale is numerical, and categories are comparable to the ECTS scale A-F: 12; 10; 7; 4; 02; and 00, −03 are the equivalent of A; B; C; D; E; and F.

## Discussion

### Principal Findings

Changing from Tiered grades to Pass/Fail assessment for exams in undergraduate medical education during the 4th and 5th years increased the number of students failing exams. This difference leveled out when including the near-fail Tiered grade as Fail. The final academic performance did not change.

A recent meta-analysis reported limited data from the previous millennium and showed no difference in performance when comparing tiered and Pass/Fail examinations [11]. Similar results were found in more recent data reported by Ange et al [12]. Alternating assessment was recently reported to cause higher average grades when using Tiered grades, but data on the number of students passing exams were lacking [13]. Despite these uncertainties [14], a number of medical schools are transitioning to Pass/Fail grading to improve psychological well-being and satisfaction among medical students [15]. This change raises concerns about academic performance and the risk of overlooking underperforming students [16]. Our study addresses these points and may inform decision-makers on the transition from Tiered grades to Pass/Fail in medical education.

Contrary to our expectation, the number of students not passing exams was higher with Pass/Fail compared to Tiered grades. A similar trend was found in the subgroups when Pass/Fail and Tiered grade exams were alternating over the same academic year, and when looking over time. In both comparisons, more students passed the Tiered graded exams compared to the Pass/Fail exams. One possible explanation is the lack of opportunity to address underperformance with a low grade, prompting examiners to shift their focus from rewarding high achievers to ensuring standards are met by underperforming medical students. Another reason might be related to learning differences among students, in that Tiered grades might support students’ self-evaluation of performance linked with higher extrinsic motivation. However, we saw no change in the average final licensing exam grades, which suggests an absence of influence on the students’ final academic performance.

The choice of grading principles is made by the study board. The curriculum reform in 2018 changed the grading principles based on discussions on study board meetings in the year leading up to the decision, with evaluation of and input to these discussions by members of a study lead group (BM, JE, SA). Only two members of the study board were academic staff representatives of the clinical study years (2013‐2025, SA). Some clinical academic staff expressed reservations on the switch from Tiered grades to Pass/Fail, but accepted this decision following information on the discussions in the study board, providing a background for the decision. Hence, the evaluators at the 7634 exams implemented the decision on the grading principle without opposing this. Moreover, the final exams all included an external evaluator to oversee if the examination covered the learning objectives and if the grading met the standards set by the curriculum. All external evaluators were academic staff from other universities knowledgeable about the topic being tested. None of these were involved in the change in grading principles at the 4th and 5th years, and the external observation contributed to solid evaluations at the final exams, illustrating that grading principles did not influence the final academic performance by medical students.

### Strength and limitations

The study’s credibility was strengthened by the large, longitudinal dataset, crossover design, and the broad range of medical specialties included. The retrospective collection of assessment data has likely prevented observer bias, as the assessors were unaware of the present evaluation. Grading is a definite variable, and it strengthens the credibility that all assessments over the years under study were included. The internal validity was strengthened by unaltered admission criteria, teaching principles, learning objectives, and module organization throughout the study period. Still, we cannot rule out confounding factors not accounted for. The overall low failure rate in the Master’s program stands out, and it may be attributed to the fact that only medical students passing the Bachelor’s program progress into the Master’s program. The single-center design may limit the generalizability of the findings. However, medical students are high achievers across settings, and we consider the core results applicable in other settings. We used digital reporting of assessment, which may have reduced observer bias. Many medical schools and educators have transitioned to digital exams. Most of our exams included real patients with a focus on clinical reasoning and demonstration of skills, which restricted us to the use of digital reporting of grades. Extending this thought, the use of the overview of grades provided by the digital reporting of grades for proxy assessment of quality of teaching and learning inspired the present evaluation by making grades easily accessible, a distinct benefit of digital reporting of assessments.

### Conclusion

This empirically grounded evaluation of the shift between Tiered grades and Pass/Fail assessment in undergraduate medical education, utilizing a natural experiment following curriculum reform, was strengthened by the large, longitudinal dataset and crossover design.

Pass/Fail grading at exams in undergraduate medical education was related to a higher risk of failing than tiered grad exams, a difference that leveled out when including the near-fail Tiered grade as fail. The data suggested no influence on the students’ final academic performance. Thus, we argue that a shift from Tiered grades to Pass/Fail assessment causes a shift in focus from rewarding high performance to upholding standards among underperforming medical students. As Pass/Fail evaluations correlate with enhanced well-being and reduced stress compared to Tiered grade, Pass/Fail may be preferred over Tiered grade assessments in high-stakes exams.

## Abbreviations

ECTS: European Credit Transfer and Accumulation System
PBL: Problem-based learning
USMLE: United States Medical Licensing Examination
